# Reducing Attenuation Bias in Regression Analyses Involving Rating Scale Data via Psychometric Modeling

**DOI:** 10.1007/s11336-024-09967-4

**Published:** 2024-04-04

**Authors:** Cees A. W. Glas, Terrence D. Jorgensen, Debby ten Hove

**Affiliations:** 1 University of Twente, Enschede, The Netherlands; 2 Research Institute of Child Development and Education, University of Amsterdam, Amsterdam, The Netherlands; 3 Section of Educational Sciences, Faculty of Behavioural and Movement Sciences, LEARN! Research Institute, Vrije Universiteit Amsterdam, Amsterdam, The Netherlands

**Keywords:** generalizability theory, item response theory, hierarchical linear models, disattenuation, generalized partial credit model, generalizability coefficients

## Abstract

Many studies in fields such as psychology and educational sciences obtain information about attributes of subjects through observational studies, in which raters score subjects using multiple-item rating scales. Error variance due to measurement effects, such as items and raters, attenuate the regression coefficients and lower the power of (hierarchical) linear models. A modeling procedure is discussed to reduce the attenuation. The procedure consists of (1) an item response theory (IRT) model to map the discrete item responses to a continuous latent scale and (2) a generalizability theory (GT) model to separate the variance in the latent measurement into variance components of interest and nuisance variance components. It will be shown how measurements obtained from this mixture of IRT and GT models can be embedded in (hierarchical) linear models, both as predictor or criterion variables, such that error variance due to nuisance effects are partialled out. Using examples from the field of educational measurement, it is shown how general-purpose software can be used to implement the modeling procedure.

Many studies in fields such as psychology and educational sciences obtain information about attributes of subjects through observational studies, in which raters score subjects using multiple-item rating scales. Usually, the items are assumed to measure a unidimensional latent variable (denoted by $\theta$), though multidimensional generalizations exist. Examples that we consider in this article are instruments measuring attributes of teachers such as efficient classroom management (e.g., Van der Scheer et al., 2019), instructional skills (e.g., Van der Scheer et al., 2017; Bijlsma et al., 2022), and differential instruction (e.g., Faber et al., 2018).

Observation instruments are typically comprised of polytomously scored items, such as Likert-type scales. Dichotomously scored items are a special case. Data collected using multiple-item rating scales are often analyzed using item response theory (IRT) models (van der Linden, 2016). There are several advantages of using an IRT model over analyzing scores obtained by aggregating item responses. IRT separates the effects of items and person in the response data. This so-called parameter separation supports comparison of measures on different, though linked, instruments, easy handling of missing data, including planned missingness by design, optimal item administration designs and heteroscedastic definitions of measurement error. IRT offers the possibility to explain differences in tests scores across individuals who took (partly) different tests, without these differences being affected by test- (or item-)specific effects.

Next to item-related effects, observations may contain rater-related effects and effects due to other measurement-facets, such as the specific tasks administered or the time-points of administration. Such measurement effects are often considered a nuisance because they add noise to the estimates of the attributes of the objects of measurement. Note that in this article the terms objects, objects of measurement and subjects are used interchangeably, they refer to the target of the measurement, say an essay of a student, the proficiency of a teacher, etc. Estimating a so-called generalizability theory (GT) model allows to disentangle the total variation in observations into variation due to differences across the objects of measurement (e.g., subjects) and variation due to measurement error facets (e.g., raters, occasions; Cronbachet al., 1963; Brennan, 2001). Using the estimates of the variance components, the impact of measurement error on the target of interest scores can be assessed (Cronbach and Shavelson, 2014). Further, generalizability coefficients computed from these variance components can be used to index the (relative) magnitude of the effect of the sampled measurement occasions on the observed scores of the objects of measurement. These coefficients express the degree to which observed scores can be generalized over the measurement occasions (e.g., Fan & Sun 2014, Vispoel et al., 2018) and can be adapted to assess specific types of reliability, like test–retest and inter-rater reliability (e.g., Brennan, 2001, Vispoel et al., 2019, Shrout & Fleiss 1979, McGraw & Wong, 1996, Ten Hove et al., 2021, 2022).

The combination of an IRT and GT model (labeled the GT-IRT model) provides a powerful framework for the analysis of ratings obtained by itemized instruments (Patz et al., 2002; Glas, 2012; Choi and Wilson, 2018; Shin et al., 2019; Huang and Cai, 2023). In this article, it will be shown how the GT-IRT model can be used to correct for measurement error in an integrated approach—for instance, how the GT-IRT model can be used to disattenuate regression coefficients in linear models. The problem that needs to be tackled is that the advantages of IRT outlined above come with a price. The GT model, and also the possible multilevel models in which the GT model may be embedded, uses the latent variables $\theta$ of IRT as dependent or independent variables rather than directly observed variables or functions of directly observed variables (e.g., scale composites). However, these latent IRT variables are estimates rather than direct observations, and the sampling variance of the $\theta$ estimates must be taken into account. One way of dealing with the problem is using plausible value imputation (see, for instance, Khorramdel et al., 2020). One of the issues is that the measurement model and structural model (the linear model on the latent person parameters) must be estimated jointly, and plausible values would not help if they come from just the measurement model alone and therefore do not have the correct relationship with the other variables. In the present article, an extension of this approach is used, where the plausible values are the full set of samples from a Markov chain Monte Carlo (MCMC) estimation procedure for a fully Bayesian model (see, for instance, Fox & Glas, 2001, 2003).

This article is structured as follows. First, we present the IRT model to map the discrete item responses to a continuous latent scale, and the GT model to separate the variance in this latent measurement into variance components of interest and variance components of nuisance. Generalizability coefficients to assess the measurements’ reliability and agreement among raters are also discussed. Second, a Bayesian estimation procedure that allows for the estimation of the model parameters in an integrated approach is outlined. Third, using examples from the field of educational measurement, it is shown how the GT-IRT model can be embedded in linear regression and factor analysis models, that incorporate the latent variables associated with the variance of interest, and how this procedure can be used to answer substantive research questions.

## Theoretical Background

In this section, we consider a straightforward case with a fully crossed design. Generalizations to observational designs that are not fully crossed will be treated in the example sections. We outline the model for a two-faceted observational design where raters (i.e., the first measurement facet, indexed $r = 1,\ldots ,R$) assess objects (e.g., work by students, lessons delivered by teachers; indexed $j = 1,\ldots ,N$) on a second measurement facet (e.g., tasks, time-points; indexed $t = 1,\ldots ,T$) using an instrument with *K* items (indexed $k = 1,\ldots ,K$) to provide information about an attribute of the objects (e.g., teacher proficiency or creativity). It is assumed that each item *k* has $M_k + 1$ response categories labeled $m = 0,\ldots ,M_k$ and that the instances of both measurement facets (i.e., the raters and time-points) are randomly sampled from a larger pool of potential instances of these facets.

To put the presentation of the GT-IRT model in perspective, consider the setup of the first example that will be presented in the section *Empirical Examples*. The example pertains to the evaluation of changes in the instructional skills of 34 teachers after they participated in an intensive data-based decision making intervention (Van der Scheer et al., 2017). The teachers were recorded three times prior to the intervention, and three times after the intervention, and all recordings were assessed by four independent raters. Instructional skills were measured with an observation instrument of 35 Likert-scale items with 4 response categories. So in this example, $N=34$, $R=4$, $T=6$, $K=35$ and $M_k = 3$, for all *k*.

Note that this setup could also be viewed as a 3-facet $j \times r \times t \times k$ design. However, in IRT models and their applications, such as in large-scale educational surveys, the items making up a test are usually treated as a fixed facet, where no generalization to a larger population of possible items is involved. For simplicity, in subsequent steps we drop the item subscript *k* from the GT part of the model. Interactions of item effects with other facets of measurement are still reflected in the GT-IRT model. For instance, if an incomplete item administration design is used, item parameters can still vary across other measurement facets (*r* and *t*). The possibilities of viewing items as randomly sampled from some domain will be returned to in the Conclusions section.

### IRT Model

The IRT model is used to map the discrete item responses onto a continuous latent scale and to model both item and subject parameters. The IRT model used here is the generalized partial credit model (GPCM; Muraki, 1992) in a logistic representation. This choice is not essential. Verhelst et al. (1997) have shown that the GPCM generally yields results that are similar to those obtained by the graded response model (Samejima, 1969) or the sequential model (Tutz, 1990). The reason is that their item response curves, relating observed responses and the latent variable $\theta$, are very close. The same holds for the logistic and normal-ogive (or probit) representation of these models.

The measurement occasion pertains to the observation of the target of measurement *j* and one or more facets that influence the observation, say, a rater *r*, and a time-point *t*. In the GPCM, the probability of a response $Y_{jrtk}$ in categories $m = 0,\ldots ,M_k$ is given by

1 $$\begin{aligned} P\left( Y_{jrtk}=m\big |\theta _{jrt}\right) = \frac{\exp \left( \sum _{h=1}^{m} \alpha _k (\theta _{jrt}-\delta _{kh})\right) }{1\ +\sum _{y=1}^{M_k}\exp \left( \sum _{h=1}^{y} \alpha _k (\theta _{jrt}-\delta _{kh})\right) }, \end{aligned}$$

where $\alpha _k$ is the discrimination parameter of item *k*, $\delta _{kh}$ are item-location parameters, and $\theta _{jrt}$ is the latent variable associated with measurement occasion *jrt*. It is assumed that $\delta _{k0} = 0$ and summations with upper-bounds and lower-bounds equal to zero yield a zero result. Note that the denominator is a normalization factor, that is, it is the sum over all response categories to ensure that the probability of all possible responses equals one. As a consequence, the probability of a score in the zero-category is given by

2 $$\begin{aligned} P\left( Y_{jrtk}=0 \big |\theta _{jrt}\right) = \frac{1}{1\ +\sum _{y=1}^{M_k}\exp \left( \sum _{h=1}^{y} \alpha _k (\theta _{jrt}-\delta _{kh})\right) }. \end{aligned}$$

This general formulation can be further extended to more facets (other than items), by adding more subscripts to the latent variable $\theta _{jrt}$.

### GT Model

The GT model is used to separate the measurement variance into variance components of interest and nuisance components, based on the different facets of the measurement design. Because the IRT model in Formula (1) is a probability model that already accounts for the item-specific effects on the uncertainty of the latent scores and eliminates item-specific random error, the GT variance decomposition concerns the latent observations $\theta _{jrt}$.

The measure of instructional skills of teacher *j* by rater *r* at time-point *t*, $\theta _{jrt}$ is decomposed into the main effect of the object of measurement (the teacher) and measurement facets, that is,

3 $$\begin{aligned} \theta _{jrt}=\theta _{1j}+\tau _{2r}+\tau _{3t} +\tau _{4jr}+\tau _{5jt}+\tau _{6rt}+\epsilon _{jrt}, \end{aligned}$$

where $\theta _{1j}$, $\tau _{2r}$, and $\tau _{3t}$ are the main effects of teacher $j, j=1,..,N$, rater $r, r=1,..R$, and time-point $t, t=1,..,T$, respectively. Further, $\tau _{4jr}$, $\tau _{5jt}$, and $\tau _{6rt}$, are the two-way interaction effects between subject *j* and rater *r*, and time-point *t*, respectively. $\epsilon _{jrt}$ is the three-way interaction effect between subject *j*, rater *r*, and time-point *k*, which is confounded with the random error.

In the examples given below, the variable $\theta _{1j}$ will be used in a mixture of the GT model with a linear regressions model to reduce attenuation. In the next section, the model will also be used to construct generalizability coefficients, using the following variance decomposition. The total variance of $\theta _{jrt}$ is decomposed into orthogonal variance components associated with each effect, that is,

4 $$\begin{aligned} \sigma _{\theta _{jrt}}^2=\sigma _{1j}^2+\sigma _{2r}^2+\sigma _{3t}^2 +\sigma _{4jr}^2+\sigma _{5jt}^2+\sigma _{6rt}^2+\sigma _{7jrt}^2. \end{aligned}$$

This general variance decomposition model can be applied in many situations. Raters may be crossed with all measurement occasions or may be distributed over measurement occasions according to some linked design (see Empirical Example 2). Facets may be random (drawn from a pool of possible instances) or fixed (when the possible tasks making up a facet are limited). Further, the ratings may pertain to an absolute judgement (where agreement of raters is relevant) or a relative judgement (pertaining to the ordering of objects where consistency across raters is relevant).

### Reliability and Agreement in the GT-IRT Model

Based on the GT-IRT model defined in the previous sections, generalizability coefficients are summary statistics that express the reliability of ordering subjects and are of interest for correlation studies and linear regression analysis. These coefficients also offer the opportunity to assess the reliability of the absolute standings of subjects on an attribute, such as a latent score on a diagnostic test. This is accomplished using indices of dependability, which are closely related to intraclass correlation coefficients (ICCs) for agreement. The difference will be discussed below.

Bechger et al. (2003) point out that the concepts of reliability in classical test theory (CTT) and IRT are very much alike. Both are derived from the variance decomposition $var(\theta ) = var(E(\theta | y)) + E(var(\theta | y ))$, where $\theta$ is a true score or latent person variable, $var(\theta )$ is the population variance of $\theta$ and $E(\theta | y)$ is the expectation of $\theta$ given the observations *y*. Reliability is expressed as a variance ratio, that is,

5 $$\begin{aligned} \rho ^2 = 1 - \frac{E(var(\theta | y ))}{var(\theta )} = \frac{var(E(\theta | y))}{var(\theta )}. \end{aligned}$$

The true score $\theta$ is unknown, so an estimator is plugged in. In IRT, this leads to the so-called expected a posteriori (EAP) score. Note that $var(E(\theta |y))$ is the variance of the EAP estimates over the complete sample of responses. The measurement error on the latent scale, that is $E(var(\theta | y ))$, consists of two elements: the uncertainty regarding the position of $\theta$ on the latent scale given the response pattern *y* and the uncertainty modeled by a GT model.

Besides being an easy summary of the generalizability of the assessments, generalizability coefficients also support a so-called design study (D-study). In this approach, the variance components of the GT model are estimated first in a so-called generalizability study (G-study), and subsequently the results are used in a D-study to estimate the number of raters, time-points and other possible facets to obtain a certain target reliability level. To define generalizability coefficients that facilitate this, an EAP estimator is defined that is analogous to the test score used in CTT and its’ extension, generalizability theory, which is $S_j = \sum _{r,t} Y_{jrt} /{RT}$, where $Y_{jrt}$ is a manifest continuous observation or total score. So we take the average over raters and time-points, that is $S_j = \sum _{r,t} \theta _{jrt} /{RT}$. After decomposing the variance of $S_j$ in a GT-IRT model, in the D-study the desired reliability of an assessment averaged over raters and time-points can be estimated by varying the number of raters *R* and the number of time-points *T* in

6 $$\begin{aligned} \rho ^2_{R}=\frac{\sigma _{1j}^2}{\sigma _{1j}^2+{\sigma _{4jr}^2/R}+{\sigma _{5jt}^2/T}+{\sigma _{7jrt}^2/(RT)}}, \end{aligned}$$

with $\sigma _{1j}^2 = \text {Var}(E(S_j | \textbf{Y}_j ))$ and vector $\textbf{Y}_j$ is the concatenation of all responses given regarding teacher *j*. The other variance components are posterior variances with an analogous definition, for instance, $\sigma _{2r}^2 = \text {Var}( \tau _{2r} | \textbf{Y}_j )$. Note that Formula (6) is analogous to the expression for reliability in the CTT-version of GT, only the variance components are defined differently. Note further that the three variance components present in Formula  (4), that is, $\sigma _{2r}^2$, $\sigma _{3t}^2$ and $\sigma _{6rt}^2$, do not appear in the denominator of Formula  (6). The reason is that the assessment is averaged over time-points and raters, so it works out the same for all objects in the sense that their ordering is not affected by these factors. If the interest is in an absolute assessment rather than a relative assessment, the assessments of the various raters on various occasions must be as similar as possible. Then, the three omitted variances become important and must be included in the denominator of the coefficient. This leads to a coefficient of Agreement given by

7 $$\begin{aligned} \rho ^2_{A}=\frac{\sigma _{1j}^2}{\sigma _{1j}^2+{\sigma _{2r}^2/R} + {\sigma _{3t}^2/T} + {\sigma _{4jr}^2/R}+{\sigma _{5jt}^2/T}+ {\sigma _{6rt}^2/(RT)}+{\sigma _{7jrt}^2/(RT)}}. \end{aligned}$$

The variance components are posterior variances defined analogously to the variance components in Formula (6). Again, the expression for Agreement is analogous to the expression in the CTT-version of GT, but with a different definition of the variance components.

In IRT, two versions of reliability are distinguished: global reliability and local reliability. Global reliability refers to the concept as it is used in CTT, say, the extent to which two randomly chosen persons from some population can be distinguished, either in the available sample, or in circumstances with the same number, but other raters and time-points sampled from their respective populations, available. Local reliability is defined locally on the latent scale and refers to the precision with which $\theta _j$ is placed on the $\theta$-scale. Local reliability is, for instance, of interest for assessing the probability that a person is above or below some cut-off point on the latent scale, or for assessing to what extent two latent scores $\theta _j$ and $\theta _{j'}$ can be distinguished. The value of a person’s latent variable can be estimated by its posterior expectation and the precision of the estimate can be represented by the associated posterior variance. If the measurement involves raters and time-points, the introduction of a GT model on $\theta$ follows the same lines as above. However, the variance component in the numerator now only pertains to one specific person *j*. Therefore, the numerator of Formula (5) becomes $var_j(E(S_j|\textbf{Y}_j))$, and the denominator is adapted accordingly.

### Estimation

In the examples, concurrent estimates are made for three integrated models: an IRT model, a GT model and a linear model.

First, we need to find a suitable IRT model. As already mentioned above, IRT models have many advantages but these advantages only apply when two conditions are met: the IRT model must fit the data, and when linking the model via the latent variable $\theta$ to a GT model, or any other regression model for that matter, the variance in the estimates of the latent variables must be properly taken into account. Starting with the requirement of model fit, it must at least be shown that the item parameters apply to all sub-populations (say all raters, all time-points, etc.) and that the item response probabilities given by the formulas 1 and 2 as a function of $\theta _{jrt}$ lead to a reasonable representation of the data in all these sub-populations. The latter is known as the requirement of no differential item functioning. Two approaches are possible to evaluate these requirements. The first is to run a separate analysis in a frequentist framework, say a marginal maximum likelihood (MML) framework and use the by now quite comprehensive collection of fit indices available in such a framework (see, for instance, Glas, 2005). The second approach is to evaluate the requirements in the Bayesian framework used for the concurrent estimation of the GT-IRT model (see, for instance, Fox, 2010, Levy & Mislevy, 2016). Though testing the IRT model is an essential first step, it is beyond the scope of the present article.

The next step is to obtain a concurrent estimate of the parameters of the GT-IRT model and the regression model. At least two options are open: an MML procedure or a Bayesian approach. A drawback of the MML approach is that the complex dependency structures in the data requires the evaluation of various nested integrals (see, for instance, Fox & Glas, 2001, p. 287). A fully Bayesian approach does not have that drawback. An MCMC procedure can be used to generate the posterior distributions of all parameters. From these distributions, point estimates can be obtained from their posterior means or medians. The repeated draws created in the procedure are equivalent to a huge set of plausible values. One of the nice things about Bayesian estimation using MCMC computational methods is that functions of parameters can be sampled along with their constituent parameters. Therefore, the computation of credibility regions for the generalizability coefficients, which are functions of variances, is relatively straightforward. Further, a Bayesian approach allows to incorporate prior beliefs about the distribution of model parameters, which eases estimation of, e.g., variance components for small sample sizes (which is often the case in observational studies). More common is to use vague and uninformative priors and this approach will be followed in the examples. Further explanation will follow below.

One of the important issues when using an MCMC procedure is whether the Markov chain has actually converged. There are many tools available for checking convergence, but that topic will not be extensively discussed in the present article. All examples were computed using 60,000 iterations with 5,000 burn in iterations, which proved to be more than sufficient for convergence, judging from adequate mixing in the trace plots.

### Identification

An IRT model must be identified by fixing the origin and scale of the latent dimension. This is typically done by constraining either item parameters or the mean and variance of the distribution of latent parameters. Regarding using constraints on the item parameters, often the item parameters are already known through estimation in other applications and are entered as fixed constants. If this is not the case, the simplest way is to fix one discrimination parameter to one, and one location parameter to zero. This may, however, not be an optimal solution if the item parameters that are fixed to identify the model are poorly conditioned by the data. For instance, if a dichotomously scored item has a very small number of correct responses, the standard error of such an item is very large, this will propagate to all other item parameters, which will all become inconveniently large. The same occurs when the discrimination parameter of the item used for identification is very low or very high. Therefore, a better approach is to set the sum of the item location parameters to zero, and the product of the item discrimination parameters to one. However, depending on the priors and the MCMC computational algorithm, this approach is not straight-forward. The alternative is fixing population parameters. Without the presence of a linear model on the ability distribution, fixing its mean and variance zero and one, respectively, is the simplest approach. However, in the present framework, the scale of the latent dimension is made up of several variance components. Fixing the mean and variance of the best conditioned distribution, usually the distribution of the main effect of the objects of measurement *j*, is a practicable approach.

### Priors

Bayesian estimation using MCMC computational methods entails drawing parameters from the posterior distribution to map out its shape, where the posterior distribution is proportional to the product of likelihood of the parameters given the data and the prior distributions of the parameters. For the IRT model, several suggestions are available. For instance, Albert (1992) suggests a flat prior on positive reals for discrimination parameters and a flat prior on the real line for the difficulties. Alternatively, the discrimination parameters can also be given a log normal prior or a truncated normal prior (a normal distribution restricted to the positive reals with a mean and variance both equal to one). The latter approach was used in the examples below. In these examples, the location parameters are given normal priors with variance equal to 1.0 and means equal to the category index within the item. The latter was done to reflect the likely order of the location parameters on the latent scale.

Finally, for variances an inverse gamma distribution is usually chosen, while Fox and Glas (2001) suggest Jeffrey’s prior and discuss uniform and inverse-chi-squared priors as an alternative for small sample sizes. In the examples given below, priors for precision parameters (the reciprocal of variances, such as the variance components in a GT model) were Gamma distributions with the two parameters equal to 0.01, such that the expectation and variance of the precision were equal to 1.0 and 500, respectively. So this prior was quite vague. The covariance matrix introduced below in the last example was given an inverse Wishart distribution with an identity matrix as a parameter.

## Empirical Examples

All three examples presented below are derived from a research project regarding teachers’ instructional skills. The aim of the present section is to give some examples of the modeling possibilities of the approach presented above. For more information about the context in which the data were collected and the items used, refer to the articles mentioned in the examples. The analyses presented below are no strict replications of the data analyses in the articles: the selection of items for the analyses is different, and the results of the analyses should not be used for substantive conclusions. For such purposes, refer to the original three articles. All examples in the sequel were analyzed using both the program Open Bugs and the program JAGS (Plummer, 2017) via the R package runjags. Scripts and data files associated with this article are available on the Open Science Framework (OSF, https://osf.io/knzw9/).

### Example 1: Evaluation of Teachers’ Instructional Skills

In this example, the data from Van der Scheer et al. (2017) are used. In the research project, changes in the instructional skills of 34 teachers participating in an intensive data-based decision-making intervention were evaluated. In this example, we discuss three approaches to the estimation of IRT item parameters, G- and D-coefficients for various research designs, and results of a linear model based on the latent attribute scores issued from the GT-IRT model as outcome variables.

#### Data

The 34 teachers were recorded three times prior to the intervention, and three times after the intervention, and all recordings were rated by four independent raters. Instructional skills were measured using the so-called the ICALT (International Comparative Analysis of Learning and Teaching, Van de Grift, 2007, 2014) consisting of 35 Likert-scale items with 4 response categories.

#### Modeling Procedure

The item responses are modeled by the GPCM, so the probability model for the response by a rater *r* at time-point *t*, when judging teacher *j* in category $m=0,...,3$ on an item *k* is given by the Formulas (1) and (2).

The measurements $\theta _{jrt}$ are decomposed into main effects of the object of measurement and all effects of the measurement facets, as well as all their interaction effects. The model is given in Formula (3), and its variance decomposition is given in Formula (4). In this example, $J=34$, $R=4$, and $T=6$. Thus, there were 816 responses ($N \times T \times R = 34 \times 6 \ \times 4 = 816$) to the 34 items. To keep the tables of the present example concise, 10 out of the 34 items were randomly chosen for the analyses, yielding 8160 observed item responses for analysis.

The first step in the analysis is to establish that the IRT model fits the data. We will not go into detail here, but the items fit the model adequately, as can be inferred from the supplementary information provided with the article of Van der Scheer et al. (2017). As a next step, three approaches are considered for the estimation of the GT-IRT model.

#### Results

**Concurrent Estimates** To take all uncertainty regarding the model into account, the ideal procedure is to obtain concurrent draws of all IRT and GT parameters from their joint full posterior simultaneously. The results of this approach will be presented first. However, in many situations, this approach proves impractical, so alternatives will be presented next.

Under the heading “Concurrent all parameters”, Tables 1 and 2 give the estimates of the discrimination parameters (Table 1) and the average of the three item location parameters $\delta _{k1}$, $\delta _{k2}$ and $\delta _{k3}$ (Table 2). For every item, this average gives an indication of the overall location of the item on the latent scale. The column labeled “Median” gives the median of the MCMC generated posterior distribution and the columns labeled “L2.5%” and “U97.5%” give the boundaries of the central 95% credibility region.

MML (marginal maximum likelihood) estimates were used as starting values. These estimates were computed disregarding the linear GT model and the hierarchical structure of the data. Enhancing an IRT model in an MML framework with complicated population models proves practically infeasible due to the increased dimensionality of the latent space across which numerical integration must marginalize.

**Table 1:** Empirical Example 1: Differences between estimates of discrimination parameters using various estimation strategies.

Item	Concurrent all parameters			Empirical Bayes			Fixed values
	L2.5%	Median	U97.5%	L2.5%	Median	U97.5%	
1	2.129	2.628	3.225	2.270	2.700	3.176	3.8032
2	1.891	2.316	2.789	2.015	2.364	2.784	3.2183
3	2.182	2.695	3.264	2.260	2.772	3.383	3.8454
4	2.002	2.464	2.993	2.089	2.577	3.112	3.5996
5	0.957	1.168	1.402	0.980	1.212	1.485	1.5545
6	0.636	0.793	0.975	0.651	0.814	1.005	1.1633
7	1.481	1.810	2.182	1.542	1.898	2.241	2.6945
8	0.869	1.066	1.287	0.885	1.091	1.328	1.5424
9	0.723	0.896	1.084	0.744	0.919	1.117	1.2891
10	0.487	0.612	0.752	0.510	0.635	0.779	0.8985

**Table 2:** Empirical Example 1: Differences between estimates of average location parameters using various estimation strategies.

Item	Concurrent all parameters			Empirical Bayes			Fixed values
	L2.5%	Median	U97.5%	L2.5%	Median	U97.5%	
1	$-$ 3.048	$-$ 1.817	$-$ 0.997	$-$ 3.673	$-$ 3.218	$-$ 2.748	$-$ 3.462
2	$-$ 1.990	$-$ 0.956	$-$ 0.287	$-$ 2.628	$-$ 2.167	$-$ 1.808	$-$ 2.309
3	$-$ 2.908	$-$ 1.686	$-$ 0.860	$-$ 3.800	$-$ 3.143	$-$ 2.628	$-$ 3.358
4	$-$ 2.462	$-$ 1.338	$-$ 0.611	$-$ 3.145	$-$ 2.700	$-$ 2.319	$-$ 2.910
5	$-$ 1.260	$-$ 0.736	$-$ 0.350	$-$ 1.851	$-$ 1.372	$-$ 1.104	$-$ 1.399
6	$-$ 0.804	$-$ 0.432	$-$ 0.165	$-$ 1.068	$-$ 0.851	$-$ 0.656	$-$ 0.938
7	$-$ 1.003	$-$ 0.220	0.317	$-$ 1.553	$-$ 1.195	$-$ 0.845	$-$ 1.357
8	$-$ 0.668	$-$ 0.173	0.207	$-$ 1.002	$-$ 0.741	$-$ 0.489	$-$ 0.854
9	$-$ 0.308	0.095	0.418	$-$ 0.589	$-$ 0.375	$-$ 0.183	$-$ 0.463
10	0.109	0.382	0.598	$-$ 0.079	0.061	0.199	0.007

**IRT estimates: Empirical Bayes Estimates and Fixed Item Parameter Values** As already noted, the concurrent procedure is not always practical, for instance, if the number of item parameters is very large, or when the instrument has already been calibrated. In these situations, prior information regarding the item parameters can be incorporated in the priors (a method dubbed here empirical Bayes), or the available item parameter estimates can be used as fixed values, that is, they are used as auxiliary data.

In Tables 1 and 2, the columns labeled "Empirical Bayes" give the estimates of the item discrimination parameters and the average item location parameters using the Empirical Bayes procedure. The estimation procedure was generally similar to the concurrent procedure and also the prior for the discrimination parameter was the same as in the concurrent approach. However, the item location parameters were given a normal prior with a mean as obtained in the MML estimation step and a variance of 10.0. The last column in Tables 1 and 2 was obtained by plugging in the MML item parameter estimates as fixed constants.

It can be seen that the concurrent and empirical Bayes estimates of the discrimination parameters were quite close, but clearly lower than the fixed discrimination parameters. Still, the correlations between the estimates are always above 0.99. For the average location parameters, the picture was different, in the sense that the empirical Bayes estimates and the Fixed values were closer and both lower than the concurrent estimates. Still, the correlations between the location estimates were high, always higher than 0.98. The conclusion is that the estimates from the three methods cannot be used interchangeably, but the relations between the items given the estimation procedure are well preserved. Table 3 gives the estimates of the variance components of the GT model for the three estimation approaches. Here, the estimates of the concurrent and empirical Bayes approaches are quite close, while the imputation of fixed item parameters leads to some non-systematic deviations.

**Table 3:** Empirical Example 1: Estimates of variance components using various estimation strategies.

	Concurrent estimate			Empirical item priors			Fixed item parameters		
	L2.5%	Median	U97.5%	L2.5%	Median	U97.5%	L2.5%	Median	U97.5%
$\sigma _{2r}^2$	0.141	0.204	0.308	0.137	0.196	0.295	0.135	0.193	0.291
$\sigma _{3t}^2$	0.135	0.193	0.291	0.132	0.188	0.279	0.132	0.187	0.280
$\sigma _{4jr}^2$	0.142	0.192	0.267	0.140	0.188	0.259	0.110	0.143	0.187
$\sigma _{5jt}^2$	0.168	0.229	0.320	0.164	0.223	0.309	0.120	0.154	0.197
$\sigma _{6rt}^2$	0.119	0.166	0.239	0.119	0.165	0.237	0.116	0.159	0.228
$\sigma _{7jrt}^2$	0.292	0.387	0.537	0.278	0.373	0.512	0.169	0.201	0.239

Model identified by setting $\sigma _{1j}^2$ equal to 1.0.

**Linear Model on Latent Attribute Scores** One of the questions in this research was whether the intervention of participating in the course had an impact. The intervention took place after the first three time-points. The means and the mean difference between the first and last three measurements are given in Table 4. The column labeled "816" gives the values computed with the actual 816 response patterns that were available. Note that the values for the first three time-points are indeed lower that the values for the last three time-points. The difference between the measurements before and after the intervention are displayed in the last four rows. The row labeled "Mean" gives the posterior mean value of the difference, the rows above and below give the boundaries of the central 95% credibility region, and the last row gives the span of this region. Note that the 95% credibility region does include the value 0.00, so if one would be interested in hypothesis testing, the difference before and after the intervention would not be significant.

Next, it was investigated how many more observations would need to be available before the confidence region would no longer include 0.00. Given the estimated model parameters, additional response patters were sampled, doubling the number of response patterns three times, to obtain data sets of 1632, 3264 and 6528 response patterns. The numbers and effects of raters and time-points were not altered. In the last column of Table 4, it can be seen that an increase to 6528 does indeed lead to excluding the value 0.00 from the 95% credibility region. So in more traditional (frequentist) terms, the conclusion is that the power is then increased, such that the null hypothesis of no effect is rejected with a significance probability of 95%.

**Table 4:** Empirical Example 1: Changes in mean proficiency over subsequent measurements.

	Mean proficiency			
Time	Number of records			
Point	816	1632	3264	6528
1	0.661	0.774	0.758	0.862
2	0.438	0.532	0.555	0.583
3	0.640	0.743	0.729	0.846
4	0.779	0.895	0.886	1.048
5	0.861	0.957	0.975	1.106
6	0.858	0.958	0.979	1.093
Difference between $T=1,2,3$ and $T=4,5,6$				
L2.5%	$-$ 0.115	$-$ 0.081	$-$ 0.075	0.014
Mean	0.247	0.254	0.257	0.303
U97.5%	0.596	0.600	0.592	0.643
95% range	0.771	0.681	0.667	0.657

**Table 5:** Empirical Example 1: D-study: Agreement and reliability as a function of various numbers of raters and time-points.

			Global			Medium trait level			Low trait level		
	*R*	*T*	L2.5%	Median	U97.5%	L2.5%	Median	U97.5%	L2.5%	Median	U97.5%
$\rho ^2_{A}$	4	3	0.087	0.425	0.792	0.113	0.496	0.840	0.034	0.228	0.638
	4	6	0.140	0.538	0.848	0.176	0.609	0.885	0.055	0.318	0.725
	2	6	0.102	0.474	0.786	0.129	0.546	0.837	0.039	0.264	0.641
	4	4	0.088	0.410	0.751	0.112	0.480	0.807	0.033	0.216	0.591
	6	6	0.114	0.507	0.847	0.147	0.579	0.884	0.046	0.292	0.718
$\rho ^2_{R}$	4	3	0.881	0.917	0.943	0.902	0.937	0.959	0.659	0.819	0.903
	4	6	0.909	0.940	0.962	0.927	0.954	0.972	0.728	0.865	0.932
	2	6	0.848	0.898	0.935	0.877	0.922	0.952	0.601	0.784	0.885
	4	4	0.833	0.883	0.920	0.862	0.910	0.941	0.568	0.756	0.864
	6	6	0.917	0.944	0.964	0.932	0.958	0.974	0.745	0.875	0.937

**Generalizability Study and Design Study** In a D-study, the number of raters and time-points can be varied to investigate their relation to the expected reliability and agreement of future applications of the instrument. When the estimates of the coefficients of reliability and agreement obtained in a G-study are too low, more raters may be used to achieve a certain target reliability. Or the number of raters may be lowered for financial reasons, if the thus adjusted coefficients do not fall below the target. For the present example, a small D-study was carried out to assess the global and local reliability and agreement. In this example, we focus on the first three time-points. The reason is that expected reliability and agreement indicates that these observations will be stable under ’parallel occasions’ (i.e., interchangeable raters, interchangeable time-points). In this study, this is not the case, because of the intervention that took place after the third observation. Therefore, the point of departure are the first three time-points. The results are given in Table 5. In principle, the values of local reliability are unique for all teachers *j*. To keep the table manageable, the local values are only presented for an average scoring teacher (average observed total score summed over time-points and raters) and a low scoring teacher (lowest observed total score summed over time-points and raters). The values in the rows for $R=4$ and $T=3$ give the results for the two coefficients for the actual design of the G-study. The other rows give the values of the coefficients when either the number of raters, the number of time-points or both are varied. The boundaries of the central 95% credible limits are given in the columns labeled “L2.5%” and “U97.5%” in Table 5.

The values in the table are as expected: reliability indices are higher than agreement indices, both decrease with fewer time-points or raters, and increase if time-points and raters are increased. So more observations result in a higher reliability and agreement. Further, the estimates for the average scoring teacher are higher than those for the low scoring teacher. That is, in the extremes of the scale, the reliability and agreement drops. Finally, it can be seen that the values for the average scoring teachers are higher than the values for the global reliability and agreement. This is explained by the fact that the global values pertain to the complete sample of teachers and the whole range of score levels.

### Example 2: Differentiated Instruction

In this example, it is shown how the latent attribute scores issued by the GT-IRT model are used as a predictor variable, instead of as an outcome variable as in the previous example. The motivation for this approach is to decrease the attenuation effect, that is, a predictor’s estimated effect will be attenuated to the degree it is unreliable. So this GT-IRT model can yield a disattenuated regression coefficient, as well as the reliability estimate that quantifies how much a manifest sum-score’s effect would be attenuated.

The data are from Faber et al. (2018) who investigated the association between teachers’ differentiated instruction and their students’ changes in mathematical achievement.

#### Data

Faber et al. 2018 investigated the relationship between differentiated instruction of $N = 51$ teachers (which is the predictor) and their $S = 953$ students’ change in mathematical achievement as a criterion variable. Students’ mathematics achievement was assessed using a standardized mathematics test both as a pretest at the beginning of the school year and as a post-test at the end of the school year.

Differentiated instruction was assessed through the ICALT questionnaire. This questionnaire consisted of 35 Likert scale items with four response categories. For each of three observed lessons, the questionnaire was filled out by three independent observers. So $T = 3$, $R=3$ and $K = 35$. The research design was crossed with some missing data, that is, nine teachers were only observed twice by the three observers. Therefore, the observational data on the ICALT comprised of 432 response patterns.

#### Modeling Procedure

As in the previous example, the item responses were modeled by the GPCM, so if $\theta _{jrt}$ is the proficiency of a teacher $j (j=1,...,J)$ assessed by a rater $r, (r=1,...,R$) at time-point $t, (t=1,...,T)$, then the probability of the response on item $k, (k=1,..,K)$ for the response categories $m, m=0,...,M$, is as defined in Formulas 1 and 2. Further, $\theta _{jtr}$ is decomposed using the GT model in Formula 3, which includes all rater effects and its interactions, and the target of the measurement, which is the teacher proficiency $\theta _j$. The research question of whether the teachers’ use of differentiated instruction had an impact on the students’ change in mathematical achievement was investigated with a random intercept multilevel model.

At the student level, the researchers had access to data about students’ mathematical achievement at the end of the previous school year, grade (of 2nd- and 5th-grade students), gender, student weight, and ability group (two dummy codes, high and low). These variables were included as Level 1 covariates. The variable student weight is an administrative variable to provide a school with extra funding if a student belongs to certain disadvantaged categories. The variable ability group was derived from the relative standing of a student in a pupil monitoring system. The complete Level 1 model on the post-test score $Y_{ij}$ is given by

8 $$\begin{aligned} Y_{ij}=\beta _{0j} + \beta _1 X_{01ij} + \beta _2 X_{02ij}+\cdots +R_{ij}, \end{aligned}$$

where the residuals $R_{ij}$ are assumed independent and normally distributed. The Level 1 covariates are listed in the first 6 rows of Table 6. The Level 2 model defined on the random intercepts is given by

9 $$\begin{aligned} \beta _{0j}=\gamma _{0} +\gamma _1 \theta _j + \gamma _2 W{j} + U_{0j}, \end{aligned}$$

where the residuals $U_{0j}$ are assumed independent and normally distributed. Level 2 covariates are $\theta _j$, which is the teachers’ proficiency in using differential instruction and $W_j$ which is a variable indicating the extent to which differential instruction was planned by the teacher. In Table 6, it can be seen that $\beta _6$ and $\beta _7$ are the coefficients of a cross-level interaction between the teacher proficiency variable $\theta _j$ and the students’ ability level.

**Table 6:** Empirical Example 2: Multilevel model with ICALT as predictor.

Variable	Parameter	L2.5%	Median	U97.5%
Grade	$\beta _1$	0.712	0.888	1.068 **
Gender	$\beta _2$	$-$ 0.127	$-$ 0.075	$-$ 0.022 **
Weight	$\beta _3$	$-$ 0.073	0.005	0.083
Pre-test	$\beta _4$	0.410	0.477	0.543 **
High-ability group	$\beta _5$	0.321	0.401	0.477 **
Low-ability group	$\beta _6$	$-$ 0.302	$-$ 0.211	$-$ 0.110 **
High ability by $\theta _j$	$\beta _7$	$-$ 0.080	0.081	0.244
Low ability by $\theta _j$	$\beta _5$	$-$ 0.485	$-$ 0.145	$-$ 0.031 **
Grand mean	$\gamma _0$	$-$ 0.704	$-$ 0.583	$-$ 0.460 **
Teacher proficiency $\theta _j$	$\gamma _1$	$-$ 0.104	0.147	0.199
Planning instruction	$\gamma _2$	$-$ 0.110	$-$ 0.054	0.002
Error Level 1		0.148	0.163	0.179
Error Level 2		0.017	0.030	0.052
ICC		0.094	0.155	0.243
Main effect teachers	$\sigma _{1j}^2$	0.356	0.447	0.563
Main effect raters	$\sigma _{2r}^2$	0.151	0.222	0.345
Main effect time-points	$\sigma _{3t}^2$	0.147	0.217	0.338
Interaction teacher by rater	$\sigma _{4jr}^2$	0.164	0.224	0.312
Interaction teacher by time	$\sigma _{5jt}^2$	0.118	0.157	0.213
Interaction rater by time	$\sigma _{6rt}^2$	0.134	0.191	0.288
Error component GT model	$\sigma _{7jrt}^2$	0.140	0.185	0.247
$\rho ^2_{R}$		0.729	0.771	0.806
$\rho ^2_{A}$		0.566	0.610	0.648

#### Results

All parameters in the model, that is, all parameters of the IRT model, the GT model and the multilevel regression model were concurrently estimated using the Bayesian MCMC method discussed above. The resulting posterior medians of the regression parameters, the variance components and the intraclass correlation coefficient (ICC) which indicates the level of dependence in the multilevel model are displayed in Table 6. The first 11 rows of the table give the estimates of the fixed effects of the multilevel model, the next lines give the estimates of the variance components and the ICC. The ICC was sampled along with the other parameters. The column labeled "Median" gives the medians of the sampled posterior distributions of the parameters. An effect is assumed to be significantly different from zero if the sign of the lower and upper bound of the central 95% credibility region are the same. If this is the case, the last column has an entry with two stars.

**The Generalizability of Teaching Proficiency.** The last 8 rows of Table 6 give the estimates of the GT model. One of the issues that has to be dealt with when using a latent variable as a predictor is the scale of the latent variable. Because all other predictors are scaled to a standard normal distribution, it was decided that this was also applied to the variables $\theta _{jtr}$. The two last rows of Table 6 give the reliability and agreement of the observations. A minimum reliability coefficient of.70 is often mentioned as a norm for reliability when scores are used for low-stakes decisions, so the reliability could be considered sufficient.

**Explaining Mathematical Achievement.** The results are as follows. There is a positive effect of the grade, a small negative effect of gender (boys perform slightly better at mathematics), and a positive effect of the pretest. Note that neither teacher proficiency of using differentiated instruction $\theta _j$ nor planning differentiated instruction had a significant effect. Further, there was a negative cross-level interaction between low-ability students and teacher proficiency $\theta _j$, which of course is unfortunate: the interaction term is $-$0.145, which basically just cancels out the positive simple effect of theta (0.147), so teacher proficiency makes even less impact on low-ability students. Finally, the ICC shows that approximately 15% of the variance in the outcomes is explained by the Level 2 predictors.

Next, it was investigated whether the power of the study with respect to finding significant effects of teacher proficiency $\theta _j$ and its interaction with the two ability groups might be augmented by a possible augmentation of the reliability of the observations. Therefore, data sets were simulated with exactly the same design as the original study, using the parameters estimates obtained in the previous study, except for the variance of the error component $\sigma _{7jrt}^2$. This error component was chosen such that the reliability of the first simulation was slightly lower than the original one (0.710 versus 0.771), while the reliability of the other three replications was higher (0.861, 0.882 and 0.889). The results of the simulations are given in Table 7. Note that when the reliability is improved, the effect of the proficiency $\theta _j$ became significant. However, the interaction between the teachers’ proficiency and students belonging to the high-ability group had no effect.

**Table 7:** Empirical Example 2: Varying the reliability of the ICALT.

	L2.5%	Median	U97.5%
Reliability	0.621	0.710	0.805
$\theta _j$	$-$ 0.100	0.138	0.418
High ability by $\theta _j$	$-$ 0.088	$-$ 0.004	0.078
Low ability by $\theta _j$	$-$ 0.202	$-$ 0.101	$-$ 0.006 **
Reliability	0.861	0.882	0.898
$\theta _j$	$-$ 0.037	0.206	0.405 **
High ability by $\theta _j$	$-$ 0.043	0.032	0.102
Low ability by $\theta _j$	$-$ 0.181	$-$ 0.094	$-$ 0.017 **
Reliability	0.882	0.901	0.916
$\theta _j$	0.057	0.279	0.502 **
High ability by $\theta _j$	$-$ 0.055	0.006	0.066
Low ability by $\theta _j$	$-$ 0.144	$-$ 0.071	$-$ 0.003 **
Reliability	0.889	0.907	0.924
$\theta _j$	0.094	0.323	0.531 **
High ability by $\theta _j$	$-$ 0.044	0.008	0.064
Low ability by $\theta _j$	$-$ 0.135	$-$ 0.071	$-$ 0.007 **

### Example 3: A multidimensional GT-IRT Model

In the two previous examples, it was shown how the latent variables issued from the GT-IRT model can be embedded in a linear regression model as either outcome or predictor variables. However, the latent IRT variables need not be unidimensional. Multidimensional IRT (MIRT) models, also known as full-information factor analysis models, are also available and applied for the analysis of categorically scored item responses (Bock et al., 1988; Ackerman, 1996). The term “full-information” pertains to the fact that estimation of the model does not depend on a covariance matrix, but takes into account the complete set of observed response patterns. A Bayesian approach for the estimation of the MIRT model was presented by Béguin and Glas (2001). In the present section, an example will be given of how a GT-MIRT model can be used to take measurement error into account when making inferences about the dependence structure of response data.

The example is based on data from Dobbelaer (2019). This example pertains to observations of lessons by each of three types of assessors: the teachers giving the lessons, their students and external observers form the Inspectorate of Education. The original research interest was in the relation between the assessments of the three types of observers. In this section, various models for assessing this relation are addressed. All three types of observers used the so-called Impact! instrument. For information on the Impact! instrument and the research settings, refer to Dobbelaer (2019). The selection of data in the present article is somewhat different from the selections made in the referenced thesis. As is the case with the other examples, the present article does not invite substantive conclusions; our interest is solely in demonstrating the psychometric approach.

#### Data

For each of 25 teachers, three lessons were assessed by the three types of observers. The observations of the external observers were collected in a fully crossed design, that is, three trained raters assessed all lessons. The number of students varied from 20 to 30 students per teacher. The Impact! instrument consisted of 15 Likert-type items, with four ordered score categories per item.

#### Modeling Procedure and Results

Teaching quality is operationalized as latent variable. Define $\theta _{1j}$, $\theta _{2j}$ and $\theta _{3j}$ as the Teaching Quality of teacher *j* assessed by the external observers, the students, and the teachers, respectively. It will be assumed that the three assessments have a multivariate normal distribution, that is,

$$\begin{aligned} \begin{bmatrix} \theta _{1j} \\ \theta _{2j} \\ \theta _{3j} \end{bmatrix} \sim N \left( \begin{bmatrix} \mu _{1} \\ \mu _{2} \\ \mu _{3} \end{bmatrix} \begin{bmatrix} \sigma _{11}^2 &{} \sigma _{12} &{}\sigma _{13} \\ \sigma _{21} &{} \sigma _{22}^2 &{}\sigma _{23} \\ \sigma _{31} &{} \sigma _{23} &{}\sigma _{33}^2 \end{bmatrix} \right) . \end{aligned}$$

The items had response categories labeled $m=0,...,3$, and the responses were modeled by the GPCM, that is, (1) and (2) define the probability of a response in category *m* on item *k*. Below, two cases will be considered: one where the instrument functioned exactly the same for all types of observers, i.e., the item parameters are the same across observers, and one where the three groups of observers each have their own set of item parameters. The measurement error models for the three types of observers are as follows.

The model for the *external observers* is the GT model as defined by Formula (3), that is,

10 $$\begin{aligned} \theta _{1jrt}=\theta _{1j}+\tau _{2r}+\tau _{3t}+\tau _{4jr}+\tau _{5jt}+\tau _{6rt}+\epsilon _{7jrt}, \end{aligned}$$

for teachers $j=1,..,25$, lessons $t=1,..,3$, and external observers $r=1,..,3$. The variance decomposition is as in Formula (4), but the variance of the teacher proficiency $\theta _{1j}$ is now equal to the first element $\sigma _{11}^2$ of the covariance matrix.

In this example, *students* are nested within teachers. In such a (partially) nested—multilevel—design, some of the measurement facets are nested, resulting in the confounding of some of the effects in Formula 3. A different GT model therefore applies. The rater effects $\tau _r$ are indistinguishable from the two-way interaction effect between subjects and raters, $\tau _{jr}$. Let *i* : *j* denote that student (the rater) *i* is nested within teacher *j*. The number of students present at the lessons varies slightly, so let $N_{jt}$ be the number of students present at lesson *t*. Then the GT-model for the students indexed $i = 1,...N_{jt}$ becomes

11 $$\begin{aligned} \theta _{2(i:j)t}=\theta _{2j}+\omega _t+\omega _{i:j}\ +\omega _{jt}+\epsilon _{(i:j)t}. \end{aligned}$$

The variance of $\theta _{2j}$ is equal to the second diagonal element of the covariance matrix, $\sigma _{22}^2$.

Assume that $\theta _{3jt}$ is the latent variable associated with the self-assessment of teacher *j* regarding lesson *t*. Then it is assumed that this latent variable has a normal distribution, that is,

12 $$\begin{aligned} \theta _{3jt} = \theta _{3j} + \epsilon _{3jt} ~,~~~\epsilon _{3jt} \sim N\left( 0,\sigma _{2jt}^2\right) . \end{aligned}$$

Note that the variance of $\theta _{3j}$ is the third diagonal element $\sigma _{33}^2$ of the covariance matrix. In terms of MIRT, the model presented here is a "between-items" multidimensional IRT model. That is, every item loads on a specific latent dimension. An alternative is a so-called within-items multidimensional IRT model, where every item can load on one, more than one or all dimensions. Such models are not considered here.

**Table 8:** Empirical Example 3: Summary of models used for analyses.

Label	GT Model	IRT Model	$dim(\theta )$	$dim(\alpha ,\beta )$	N.Pars	DIC
GT.IRT1.AB1	Yes	Yes	1	1	78	38070
GT.IRT3.AB1	Yes	Yes	3	1	77	37990
GT.IRT1.AB3	Yes	Yes	1	3	198	36090
GT.IRT3.AB3	Yes	Yes	3	3	197	36080
IRT3.AB1	No	Yes	3	1	63	38110
IRT3.AB3	No	Yes	3	3	183	36070
GT.KTT	Yes	No	0	0		–

**Testing the GT-MIRT model** Four versions of the GT-MIRT model outlined in the previous section will be tested against each other. Each model was estimated by a concurrent Bayesian MCMC method. An overview of the models is given in the first four rows of Table 8. The model labeled **GT.IRT1.AB1** has only one IRT scale related to the variables $\theta _{1j}$, $\theta _{2j}$, and $\theta _{3j}$ and all responses are modeled by the same IRT model. Further, every type of observer has a distinct normal distribution for the proficiency parameters. So the model is similar to the one introduced above, except that the covariance matrix of $\theta _{1j}$, $\theta _{2j}$, and $\theta _{3j}$ is now diagonal. To identify the model, the distribution for the parameters for the students, $\theta _{2j}$, was set equal to standard normal. For the external observers’ responses, the estimation procedure resulted in $\hat{\mu }_1=-0.49$ and $\hat{\sigma }_{7jrt}^2 = 0.69$. So the external observers were far less favorable and more homogeneous regarding the proficiency of the teachers. For the self-assessments of the teachers, the estimates were equal to $\hat{\mu }_3=0.11$) and $\hat{\sigma }_{3jt}^2 = 0.84$. So these assessments were on average the most favorable. The DIC obtained is displayed in the last column as 38070. The number of parameters is determined as follows. The IRT model has 15 discrimination parameters (or factor loadings) and 45 item location parameters, so in total 60 item parameters. The parameters for the model for the external observers are the variances of the main effects, the two-way interaction effects, and the error component. This leads to 7 parameters. The model for the students and the teachers has 5 and 2 variance parameters, respectively. Finally, there are 2 free means and 2 free variances of teachers proficiency. So in total, the model has 78 free parameters.

**Generalizations of the IRT-GT model** Next, the model **GT.IRT1.AB1** is generalized in three directions. The first model with labeled **GT.IRT3.AB1** has three correlated proficiency dimensions where the item parameters are assumed to be the same over the three dimensions. In these models, we assume that the item parameters apply to all three types of observers simultaneously. The second model, labeled **GT.IRT3.AB3** is more general, since it is assumed that every type of observer has its own set of item parameters. This also holds for the third model, labeled **GT.IRT1.AB3**, but here it is assumed that the latent proficiency variable is unidimensional. That is, all items load on the same unidimensional IRT scale, but the values of the item parameters are different for the three types of observers. Further, every type of observer has a distinct normal distribution for the proficiency parameters, but the proficiency parameters load on the same unidimensional scale.

To determine the number of parameters, note that the models **GT.IRT3.AB1** and **GT.IRT3** **.AB3** have three correlated proficiency dimensions with a joint multivariate normal distribution with 3 means and a covariance matrix with 6 parameters. The origin and scale of the three dimensions need to be fixed to identify the model. In a standard multidimensional IRT model, this is usually done by fixing the three means to zero and the diagonal of the covariance matrix to one. The covariance matrix then becomes a correlation matrix. In the present model, things are a bit more complicated, because for **GT.IRT3.AB1** and **GT.IRT3.AB3** the assumption that the three types of observers have the same means and variances on the latent scale is unrealistic. Therefore, the estimates of the means and the variances of the GT part of model **GT.IRT1.AB1** were plugged in as fixed constants to identify the models **GT.IRT3.AB1** and **GT.IRT3.AB3**. The fit of the model does not depend on the chosen 6 restrictions, other restrictions on the mean and covariance would be just as valid, and the DIC used below does not depend on the values that are actually chosen. Model **GT.IRT3.AB1** has 60 item parameters, 14 parameters stemming from the GT models, 3 mean parameters and 6 parameters in the covariance matrix, minus 6 restrictions. This amounts to a total of 77 free parameters. The DIC obtained is displayed in the last column as 37990. The relative fit of the models **GT.IRT1.AB1** and **GT.IRT3.AB1** can be tested using their DIC estimates, Models with a smaller DIC should be preferred to models with larger DIC. The numbers of free parameters of the two models are approximately the same, but also the values of their DICs are quite close, though the 3-dimensional model seems to fit slightly better than the one-dimensional one. To investigate model fit further, these two models will be compared to two more general models **GT.IRT1.AB3** and **GT.IRT3.AB3**, respectively.

The previous analyses were based on the assumption that the item parameters apply to all three types of observers simultaneously. In the next two analyses this assumption is replaced by the assumption that every type of observer has its own set of item parameters. This leads to an extra of 2 times 60 item parameters. So the model **GT.IRT1.AB1** changes to **GT.IRT1.AB3** and the number of free parameters changes from 78 to 198. In the same manner, the model **GT.IRT3.AB1** changes to **GT.IRT3.AB3** and the number of free parameters changes from 77 to 197. The values for the means and variances in the covariance matrix used above were again plugged in as fixed constants. In the last column of Table 8, it can be seen that the difference between the DIC for the original models and the enhanced models is equal to 1910, so the latter two models are an important improvement. Finally, to decide between the two models, the estimates of the item parameters are inspected. Table 9 gives an overview of the correlations between the item parameter estimates of **GT.IRT3.AB1** and the three dimensions of **GT.IRT3.AB3**, which are labeled **GT.IRT3.AB3-1**, **GT.IRT3.AB3-2**, and **GT.IRT3.AB3-3**. Note that the correlation of the discrimination parameters between **GT.IRT3.AB3-2** and **GT.IRT3.AB3-1** is 0.0677, which is quite low. Also the correlation between the location parameters between first and second dimension and between the second and third dimension are low. So the conclusion is that assuming that these item parameters differ between types of observers is corroborated, and the final conclusion is that the model **GT.IRT3.AB3** fits the data best.

**Table 9:** Empirical Example 3: Correlation between item parameter estimates for two models.

	Model	GT.IRT3.AB1	GT.IRT3.AB3-1	GT.IRT3.AB3-2
$\alpha$	GT.IRT3.AB3-1	0.945		
	GT.IRT3.AB3-2	0.845	0.677	
	GT.IRT3.AB3-3	0.929	0.843	0.872
$\delta$	GT.IRT3.AB3-1	0.977		
	GT.IRT3.AB3-2	0.704	0.539	
	GT.IRT3.AB3-3	0.539	0.884	0.607

**Some Further Analyses** Combining an IRT model with a GT model is not yet the standard approach to the analysis of data from observations. Usually, the GT model is directly imposed on the observed sum scores. Therefore, it was investigated whether imposing a 3-dimensional model (one dimension for every type of observer) combined with the GT models defined above directly on observed responses rather than on latent variables would lead to important differences. The observations were the logits of the sums of the item responses labeled 0,...,3. The model is labeled **GT.KTT**. The model parameters were again estimated using the Bayesian MCMC procedure. The estimates of the correlations between the dimensions of the three types of observers are displayed in the second column of Table 10. The third and fourth columns give the correlations obtained using the models outlined above. The results for **GT.IRT3.AB1** are quite similar to the results of **GT.KTT**. So introducing an IRT model with discrimination and location parameters does not make much difference. This would, of course, change if there would be a lot of missing responses, either by design or depending on the observers response behavior. In such cases, sum scores are less meaningful. The fourth column with the results of the **GT.IRT3.AB3** model, gives a much different picture, because all correlations increase substantially. So taking into account that the observation instrument functions quite differently for the three types of observers has a significant impact on the results.

The final two analyses relate to the question what would happen if the hierarchical structure of the data, such as the nesting of responses under observers and lessons, and the nesting of students under teachers would be ignored. To address this question, the GT model was removed from model **GT.IRT3.AB1** to produce a model labeled **IRT3.AB1**, which is a 3-dimensional IRT model with the same item parameters for all three types of raters, but without a GT model to assess the reliability of the assessments. In the same manner, the model **GT.IRT3.AB3** was stripped of the GT model, to produce a three-dimensional IRT model with three sets of item parameters, This model was labeled **IRT3.AB3**. The model parameters were again estimated using the Bayesian MCMC procedure. By comparing the estimated correlations of **GT.IRT3.AB1** with **IRT3.AB1** and of **GT.IRT3.AB3** with **IRT3.AB3** in Table 10 it can be seen that ignoring the GT model and the hierarchical structure leads to an increase of the correlations. The reason is that part of the dependence between the latent variables that can be attributed to their nested structure and ignoring this part of the dependence increases the estimate of the dependencies. So ignoring the hierarchical structure leads to unwanted bias.

**Table 10:** Empirical Example 3: Latent correlations under various models.

	Model				
Correlation	GT.KTT	GT.IRT3.AB1	GT.IRT3.AB3	IRT3.AB1	IRT3.AB3
$\sigma _{12}$	0.325	0.395	0.806	0.493	0.852
$\sigma _{13}$	0.015	0.033	0.795	0.266	0.825
$\sigma _{23}$	$-$ 0.018	$-$ 0.019	0.761	0.181	0.823

## Conclusion

The aim of this article is to draw attention to the possibility of building models for observation studies with itemized rating scales by combining an IRT model with a GT model. The two main advantages are (1) it produces an estimate of the latent variable of interest, both on the individual level and globally, which is corrected for rater and other nuisance effects and (2) the latent variables of interest can be directly embedded into linear regression models to correct for attenuation bias.

It was shown that software for Bayesian analysis using MCMC computational methods provides a flexible framework where practitioners can build their own models dedicated to their own needs. Many topics related to such analyses were ignored, for instance, checking the convergence of the MCMC chains, choosing priors and evaluation of model fit. Readers that want to dive deeper into these matters are referred to excellent books by, for instance, Lee (2007), Fox (2010) and Levy and Mislevy (2016). One point in this respect that needs mention is that the software used for this article, OpenBUGS and JAGS (via the R package runjags), sometimes fails to start up without proper starting values for item parameters and variance components. In such cases, running a simplified version of the model using MML usually works well. Another drawback of the used software is that restrictions on parameters are difficult to implement. In Example 3, it would have been helpful to identify the model via restrictions on the discrimination and location parameters, but in the used software, this proved infeasible. The solution to fix the mean and the residual variances of the GT models has no consequences for model fit, but it hampers the comparison of item parameters across analyses.

One of the important advantages of IRT is the possibility of using incomplete item administration designs. For instance, the National Assessments of primary and secondary education in the Netherlands consist of larger pools of observational items (say 320 items related to 12 tasks), a large number of students (say 2500), and a limited number of raters (say 24). The total sample of students is divided into 12 subgroups and every subgroup is administered two of the tasks in a linked design. The design for the assignment of raters to students is not fully crossed: part of the student sample is scored once by one rater, and the remainder are scored by two raters. The design of the distribution of raters across observations is linked. To complicate matters further, the difficulty of the tasks is targeted at the level of the various education streams of the students. Clearly, performing a GT study based on total scores in such a complicated design has little meaning. On the other hand, the IRT part of the GT-IRT model can account for both the design effect of (a) the combination of raters, students, and scored items and (b) the effect of the adaptive administration of tasks.

Finally, as already mentioned above, items are considered as fixed effects. That is, the itemized measurement instrument is considered as a given entity. This is in accordance with most formulations of IRT, although some IRT models with random item parameters have been proposed (see for instance Geerlings et al., 2011, and Glas et al., 2016). From the perspective of factor analysis, unidimensional IRT models with fixed item parameters are closely related to congeneric factor-analysis models, and these models have also been used in generalizability studies (see Vispoel et al., 2021).

On the other hand, the item parameters can also be seen as random (see, for instance, Jorgensen, 2021). That is, items are assumed to be sampled from a well-defined domain to which one wants to generalize, and they constitute yet another measurement facet. An advantage of this approach is that it allows for interaction between the items and the other facets. This is helpful to detect items that have large contributions to inconsistencies in response behavior. This approach is closely related to the detection of differential item functioning conditional on raters or other facets in IRT. For the one-parameter model for dichotomously scored items (Rasch, 1960), incorporating random item parameters into the GT-IRT model via a logit or probit link, is straightforward. The item response probabilities are function of a difference between the latent variable $\theta _{jrt}$ and a single item difficulty parameter $\delta _k$, that is, of $\theta _{jrt} - \delta _k$, and so the item parameter is mapped to the same latent scale as $\theta {jrt}$ and has an additive relation with the rest of the facets. For the two- and three-parameter models for dichotomously scored items, incorporating random discrimination and guessing parameters into the GT-IRT model using an analogous approach is not straightforward. For instance, the item response probabilities are a function of $\alpha _k \theta _j - \delta _k$, and the multiplication depending on both *k* and *j* presents a problem. In the same manner, for polytomously scored items, introducing random item parameters only works for the partial credit model (PCM; Masters, 1982), which is similar to the GPCM defined by Formulas (1) and (2), but without the discrimination parameter $\alpha _k$. So also here, introducing discrimination parameters again leads to difficulties.

In sum, this article showcased how a combination of IRT and GT can help improve linear modeling procedures of observation data based on itemized scales. Additionally, it was shown how different GT-based measured can be used to inspect the degree to which latent scale scores can be generalized over measurement facets such as raters and occasions. Various practical applications of the integrated model were illustrated using three empirical examples.

## Data Availability

Data, OpenBugs Scripts and the R code used in the illustration will be made available on the Open Science Framework upon publication.
